# Postpartum choriocarcinoma – a rare cause of delayed postpartum hemorrhage: Four case reports and literature review

**DOI:** 10.1097/MD.0000000000037510

**Published:** 2024-03-15

**Authors:** Guan-Lin Dai, Fu-Rong Tang, Yu Ma, Dan-Qing Wang

**Affiliations:** a Department of Obstetrics and Gynecology, West China Second University Hospital of Sichuan University, Chengdu, Sichuan, China; b Key Laboratory of Birth Defects and Related Diseases of Women and Children (Sichuan University), Ministry of Education, Chengdu, Sichuan, China.

**Keywords:** differential diagnosis, postpartum hemorrhage, puerperium, treatment

## Abstract

**Background:**

Delayed postpartum hemorrhage is rare, with an incidence of 0.5% to 2.0% in all pregnancies. The most important causes are placental remnants, infections, and placental bed subinvolution. Postpartum choriocarcinoma, a highly malignant complication of pregnancy, is a rare condition that can be easily misdiagnosed as other common causes, such as gestational remnants, and delays the diagnosis.

**Methods:**

Four patients visited our clinic complaining of delayed postpartum hemorrhage, combined with respiratory and neurological symptoms in 2 cases. Two cases were confirmed by histopathological examination and in addition, medical history, elevated human chorionic gonadotropin (hCG) level, and imaging findings help confirm the diagnosis of delayed postpartum hemorrhage caused by postpartum choriocarcinoma in other cases. Individualized combination chemotherapies were prescribed. In the light of massive cerebral metastasis in case 2, intrathecal methotrexate injection combined with whole-brain radiotherapy was prescribed.

**Results:**

Due to the absence of routine monitoring of β-hCG following full-term delivery, there was widespread metastasis at the time of diagnosis. Three patients got complete remission and there is no sign of recurrence. One patient had relapse and widespread metastasis and died at home 6 months after the last chemotherapy.

**Conclusion:**

It is important to be aware of the possibility of choriocarcinoma in patients with delayed postpartum hemorrhage. Clinicians should improve the recognition of choriocarcinoma following full-term delivery, emphasize the monitoring of β-hCG, comprehensively analyze the general condition of patients, and conduct standardized and individualized chemotherapy protocols.

## 1. Introduction

Postpartum hemorrhage is commonly defined as 500 mL of blood loss following vaginal delivery or 1000 mL following cesarean section within 24 hours postpartum. Postpartum hemorrhage is the leading cause of maternal death, accounting for 25% and arouse much concern worldwide.^[1]^ Uterine atony is the most common cause, accounting for 80% followed by gestational remnants.^[2]^ Delayed postpartum hemorrhage is rare, with an incidence of 0.5% to 2.0% in all pregnancies.^[3]^ It occurs between 24 hours and 6 weeks after delivery, without a definition of bleeding volume. Less attention is paid to late postpartum hemorrhage for clinicians and patients compared with postpartum hemorrhage that occurs within 24 hours after delivery, while it is still associated with severe anemia, shock, and even death. The most important causes of delayed postpartum hemorrhage are placental remnants, infections, and placental bed subinvolution.^[4]^ Postpartum choriocarcinoma, a highly malignant complication of pregnancy, is one of rare causes that includes pseudoaneurysm of uterine vessel and arteriovenous malformation in addition.^[5]^ Owing to its rarity and atypical manifestations, it is easily overlooked in the puerperium. A delayed diagnosis and inappropriate treatment may result in a higher incidence of relapse and resistance to chemotherapy. Moreover, choriocarcinoma following full-term delivery is associated with poor prognosis compared with choriocarcinoma following other gestational events.^[6]^ Although choriocarcinoma following full-term delivery is rare, it occurs only in 1 in 50,000 pregnancies.^[7]^ Obstetricians should be aware of the possibility of choriocarcinoma in patients with delayed postpartum hemorrhage.

Prompt recognition of the causes and appropriate treatment are critical for the management of delayed postpartum hemorrhage. Herein, we reported 4 cases of delayed postpartum hemorrhage secondary to choriocarcinoma, and widespread metastasis occurred at the time of diagnosis in 3 cases. Our aim is to assist in the differential diagnosis of the causes of delayed postpartum hemorrhage and to raise awareness of postpartum choriocarcinoma.

## 2. Case report

### 2.1. Case 1

A 36-year-old gravida 2, para 1 female was admitted to our hospital with persistent fever and irregular vaginal bleeding for 1 week. The patient underwent cesarean delivery of a healthy infant in a local hospital at 39 weeks of gestation due to placental invasion detected by ultrasound in the 36th week of gestation. Macroscopic examination of the placenta was unknown, and no histopathological examination was performed. On postpartum day 18, the patient presented with irregular vaginal bleeding, and transvaginal ultrasonography revealed intrauterine occupation. Serum β-hCG (human chorionic gonadotropin) was 158,890.25 mIU/mL (normal range < 2.0 mIU/mL). Dilation and curettage were performed but were immediately suspended due to massive vaginal bleeding. Postoperatively, chest tightness and pain appeared, combined with low fever. Chest radiography revealed a massive pleural effusion on the right side, and the patient was referred to the respiratory department. Approximately 400 mL of hemorrhagic pleural fluid was drained from the chest, which gradually reduced to approximately 200 mL every day. Respiratory symptoms were partially alleviated, but fever persisted.

**Figure 1.**
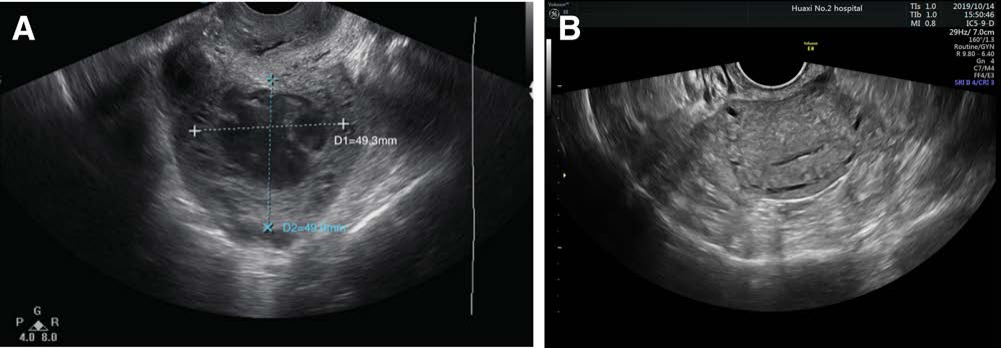
Comparison of transvaginal ultrasound. (A) A significant enlargement of corpus uteri with an occupation of 49 mm × 49 mm × 46 mm in size. Myometrium of the anterior wall was invaded with unclear boundary before chemotherapy. (B) Transvaginal ultrasound showed uterus shrunk and occupation within myometrium disappeared after chemotherapy.

**Figure 2.**
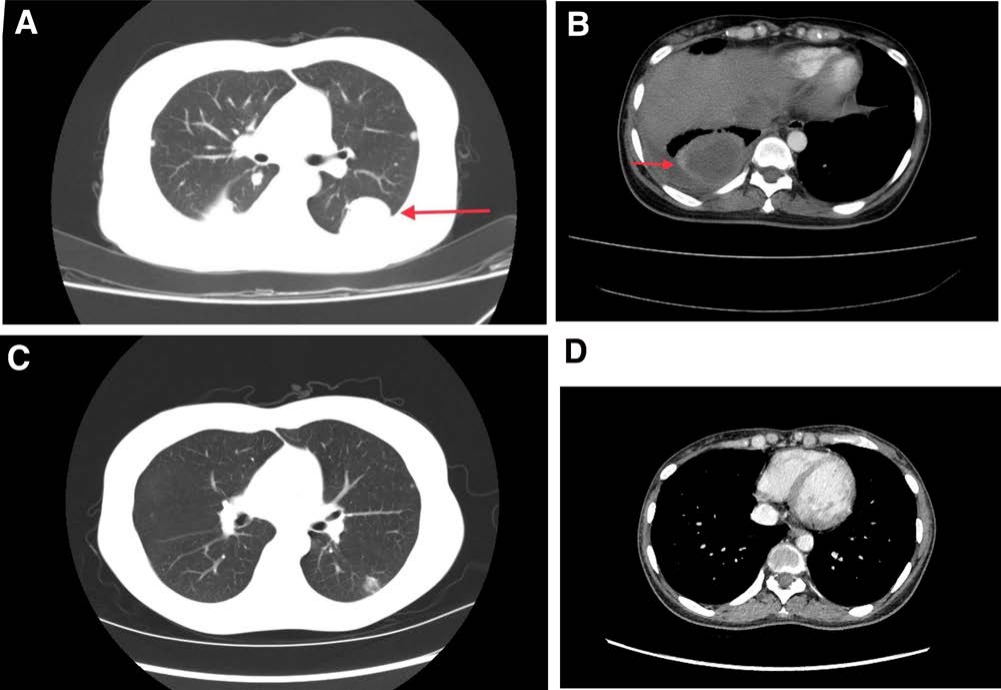
Comparison of chest CT. (A) Multiple nodules scattered in both lungs with a maximum diameter of 65 mm in left lung. (B) A mass in the right thoracic cavity, with a maximum diameter of 70 mm, is closely related to the adjacent pleura. (C) The number and size of nodules in the bilateral lung decreased after chemotherapy. (D) The mass in the right thoracic cavity disappeared after chemotherapy.

On postpartum day 25, the patient was transferred to our hospital as mentioned above. Physical examination revealed an obvious reduction of respiratory sounds in the right lung. Laboratory examination revealed an inflammatory condition. The serum β-hCG level was more than 225,000.0 mIU/mL, α-fetoprotein was 453.23 ng/mL (normal level, <8.1 ng/mL), cancer antigen was 125,242.80 U/mL (normal level, <35 U/mL) and other tumor markers were within normal ranges. The β-hCG titer of the pleural effusion was 150,695.92 mIU/mL. Other pleural effusion examination results were all within the normal range. Transvaginal ultrasound showed significant enlargement of the uterus with a maximum diameter of 71 mm and an occupation of 49 mm × 49 mm × 46 mm within the myometrium of the anterior wall of the uterus (Fig. 1A), with unclear boundaries and abundant vascular signal interiors. Computed tomography (CT) of the chest revealed multiple nodules scattered in both lungs, with a maximum diameter of 65 mm in the left lung (Fig. 2A), and a mass in the right thoracic cavity, with a maximum diameter of 70 mm, which was closely related to the adjacent pleura (Fig. 2B). CT of the brain showed no abnormalities. Postpartum choriocarcinoma with pulmonary metastasis (Federation International of Gynecology and Obstetrics stage III, World Health Organization score 9) was diagnosed.

Subsequently, chemotherapy with EMA-CO (etoposide, methotrexate, actinomycin D, cyclophosphamide, and vincristine) protocol was initiated in combination with antiinfective treatment. After 7 cycles of chemotherapy, the decline in serum β-hCG levels was unsatisfactory, and the etoposide, cisplatin/etoposide, methotrexate (MTX), and actinomycin D (EP-EMA) regimen was prescribed for subsequent treatments. Serum β-hCG normalized after the subsequent 4 cycles and an additional 4 cycles were performed. A review of the ultrasound showed that the uterus had shrunk, and the intrauterine occupation had disappeared (Fig. 1B). The number and size of the nodules in bilateral lungs decreased after chemotherapy (Fig. 2C), and the mass in the right thoracic cavity disappeared (Fig. 2D). Four years of following up shows no recurrence.

### 2.2. Case 2

A 32-year-old gravida 3, para 3 female was transferred to our hospital with irregular vaginal bleeding for 24 days, and cough and hemoptysis for 2 days. The patient successfully delivered a newborn at a local hospital and slight vaginal bleeding occurred after delivery. On postpartum day 12, vaginal bleeding increased, accompanied by dizziness and weakness. Blood transfusion and supportive treatment were administered at a local hospital and the patient underwent dilation and curettage without pathological examination. However, symptoms did not improve. Symptoms of coughing and hemoptysis appeared on postpartum day 24 and the patient was transferred to our hospital for further treatment. Review of serum β-hCG was 90,153.2 mIU/mL. Transvaginal ultrasound showed an intrauterine occupation of 86 mm × 57 mm × 56 mm in size, and pelvic magnetic resonance imaging further indicated irregular myometrial invasion that exceeded 50%. An immediate transfusion was administered for severe anemia (hemoglobin, 50.0 g/L). Although anemia improved, the symptoms of headache and dizziness worsened. Brain CT revealed a mass, 42 mm in diameter, in the left frontal lobe. Based on her medical history and imaging findings, postpartum choriocarcinoma (FIGO stage IV, WHO score 16) with lung and brain metastases was diagnosed. Multidrug chemotherapy with 5-fluorouracil and actinomycin D was administered for 7 cycles, and intrathecal MTX injection was prescribed to target brain metastasis. Headache was alleviated, and the β-hCG level in the cerebrospinal fluid decreased to 8 mIU/mL after 4 cycles of intrathecal injections of MTX. Considering the large metastatic foci in the left frontal lobe, whole-brain radiotherapy was administered. The last review of imaging examinations showed significant remission of primary and metastatic foci. There were no signs of tumor recurrence or adverse events after 6 years of follow-up.

### 2.3. Case 3

**Figure 3.**
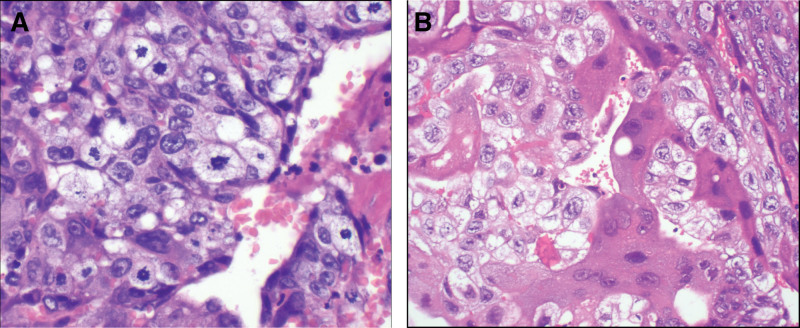
Pathological examination showed the samples from case 3 (A) and case 4 (B) were abundant in syncytiotrophoblast and cytotrophoblast that certificated the diagnosis of choriocarcinoma (magnification ×400).

A 24-year-old gravida 3, para 2 female presented to our clinic with severe vaginal bleeding. The patient successfully delivered a newborn at a local hospital and irregular vaginal bleeding persisted until postpartum day 40. During this period, a review of the transvaginal ultrasound revealed an enlarged uterus without a retained placenta or fetal membranes, and oxytocin was administered. On postpartum day 62, the patient presented to our clinic with refractory vaginal bleeding. Blood transfusions and supportive treatments were administered immediately. Emergency uterine artery embolization was performed. The serum β-hCG level was 264,000 mIU/L, and gynecologic examination revealed a 20 mm × 20 mm vaginal nodule in the anterior wall. The nodule was excised and metastatic choriocarcinoma was confirmed by histopathological examination (Fig. 3A). Based on her medical history and further imaging findings, postpartum choriocarcinoma with lung metastasis (FIGO stage III, WHO score 13) was diagnosed. A multidrug chemotherapy regimen consisting of bleomycin, etoposide, and cisplatin was administered for 6 cycles. While the tumor recurred 3 months after the last chemotherapy session, the serum β-hCG elevated to 1435.10 mIU/L. Imaging signs of recurrence were observed in both the uterus and lungs. The EMA-CO regime was subsequently prescribed for 7 cycles. Unfortunately, a second recurrence occurred 2 months later. The EP-EMA regimen was performed for 4 cycles and was transformed into the (5-fluorouracil, actinomycin D, etoposide, and vincristine) regimen for subsequent 4 cycles, followed by 4 cycles of consolidation. Unfortunately, the patient did not follow-up afterwards, and the patient died of a sudden headache at home after 6 months.

### 2.4. Case 4

A 26-year-old gravida 2, para 1 female was admitted to the hospital with sudden massive vaginal bleeding. The patient successfully delivered a newborn at a local hospital and the placenta was completely expelled. On postpartum day 20, the patient experienced massive vaginal bleeding and underwent dilation and curettage at a local hospital, without pathological examination. On postpartum day 92, heavy vaginal bleeding recurred, and transvaginal ultrasound showed a hypervascular intrauterine occupation 12 mm × 10 mm × 11 mm in size, and the muscular layer of the anterior wall was invaded with an unclear boundary. A review of serum β-hCG was more than 258,400.0 mIU/mL and decreased to 75,111.7 mIU/mL after curettage. Postpartum choriocarcinoma with deep myometrial invasion (FIGO stage I, WHO score of 5) was confirmed by histopathological (Fig. 3B) and imaging examinations. Multidrug chemotherapy according to the EMA-CO protocol was prescribed, and the serum β-hCG level normalized after 4 cycles. Three additional cycles were then performed. There was no sign of recurrence after 1 year of follow-up, and a second intrauterine pregnancy was considered 14 months after the last chemotherapy.

## 3. Discussion

Delayed postpartum hemorrhage has many causes and its clinical characteristics vary according to them. If the cause is not promptly identified and treated appropriately, severe anemia, shock, or even mortality can occur. Choriocarcinoma is a rare but malignant disease with high potential for metastasis. It can develop from various antecedent gestational events such as hydatidiform moles, abortions, ectopic pregnancies, and intrauterine pregnancies. Choriocarcinoma following full-term delivery is a rare condition,^[8]^ especially in the puerperium. Previously, the interval between the last delivery and the onset of the disease varied from 3 days to 18 years, and 47 of 123 patients were classified into the short-interval group with an interval of <4 months.^[9]^ In a retrospective study of postpartum choriocarcinoma,^[8]^ patients detected within 6 weeks. Another retrospective study reported 29 patients, of whom 12 were detected within 6 weeks.^[10]^ To the best of our knowledge, this is the first report on the differential diagnosis of delayed postpartum hemorrhage caused by choriocarcinoma and other causes of delayed postpartum hemorrhage.

Postpartum choriocarcinoma at an early stage has no specific clinical manifestations, and mostly presents with irregular vaginal bleeding. It can be easily attributed to secondary postpartum hemorrhage caused by gestational remnants and uterine atony in puerperium. Although the incidence of choriocarcinoma following full-term delivery is extremely low, it occurs in 1 in 50,000 births.^[7]^ The possibility of choriocarcinoma should be considered when delayed postpartum hemorrhage occurs. In our report, 4 patients complained of delayed postpartum hemorrhage and in 2 cases, severe anemia was observed. Three patients presented with nongynecological symptoms of metastatic foci. All patients underwent dilation and curettage, and only 2 patients underwent histopathological examination. One patient presented with refractory vaginal bleeding and underwent embolization of the uterine artery in the emergency department. All patients had elevated serum β-hCG levels, and choriocarcinoma was confirmed by pathological examination in 2 cases. Combination regimens were utilized in all patients and 1 patient with cerebral metastasis was administered an intrathecal injection of MTX combined with whole-brain radiotherapy. Three patients achieved complete remission with no signs of recurrence. One patient died from brain metastasis at home (Table 1).

**Table 1:** The clinical characteristics of patients with late postpartum hemorrhage.

Patients	Case 1	Case 2	Case 3	Case 4
Age	36	32	24	26
Reproductive history	Gravida 2, para 1	Gravida 3, para 3	Gravida 3, para 2	Gravida 2, para 1
Interval between the last delivery and onset of symptoms	18 days	12 days	Immediately	20 days
Interval between the last delivery and the diagnosis	25 days	24 days	62 days	92 days
FIGO stage	III:9	IV:16	III:13	I:5
Pathological diagnosis	NA	NA	Choriocarcinoma	Choriocarcinoma
Metastasis	Lung	Lung, brain	Vagina, lung, brain	Myometrium
Treatment	EMA-CO + EP-EMA	FA + MTX (intrathecal injection) + radiotherapy	BEP + EMA-CO + EP-EMA + FAEV	EMA-CO
Chemotherapy times	7 + 8	7 + 4	6 + 7 + 4 + 8	7
Prognosis	NED	NED	DEAD	NED and pregnancy

BEP = bleomycin, etoposide, and cisplatin, EMA-CO = etoposide, methotrexate, actinomycin D, cyclophosphamide, and vincristine, EP-EMA = etoposide, cisplatin/etoposide, methotrexate, and actinomycin D, FA = 5-fluorouracil, actinomycin D, FAEV = 5-fluorouracil, actinomycin D, and vincristine, NA = not applicable, NED = no evidence of disease.

Serum β-hCG level is the main basis for the diagnosis and monitoring of β-hCG following delivery, especially in patients with postpartum hemorrhage. Serum β-hCG levels typically normalized within 1 to 3 weeks of normal delivery. When the gestational tissue remains, serum β-hCG levels can decrease to negative levels 21 to 35 days after delivery.^[11]^ In rare cases, it can extend to 10 weeks after delivery.^[12]^ Therefore, it is critical to be aware of the possibility of choriocarcinoma in β-hCG-positive cases after excluding pregnancy. Dilation and curettage can help stop uterine bleeding and obtain biopsy specimens for histopathological examination. Typical pathological manifestations of choriocarcinomas are syncytiotrophoblasts and cytotrophoblasts with myometrial invasion. Even if the pathological examination shows no abnormalities, serum β-hCG levels should be closely monitored. In addition, transvaginal ultrasonography is the first-line examination for late postpartum hemorrhage. Typical findings of choriocarcinoma include hypervascular masses with myometrial invasion and blurred boundaries. Although the diagnosis of GTN is mainly based on clinical history and β-hCG titer, histopathological and imaging examinations can result in an accurate diagnosis at an early stage.

A previous study has reported that postpartum choriocarcinoma has a much higher tendency for widespread metastasis, particularly in the liver and brain.^[8]^ Lung is the most common site of metastasis, accounting for 64%, liver and brain account for 53.8%.^[13]^ It can also metastasize to the vagina as nodules. Patients with lung metastases commonly present with cough, hemoptysis, chest pain, and other respiratory symptoms. Headache, dizziness, and hemiplegia are the common symptoms of brain metastasis. Notably, bleeding at the site of metastasis, such as cerebral hemorrhage, can be fatal. Puerperal females commonly present with symptoms at metastatic sites as the main complaint and overlook vaginal bleeding. Their first visit is commonly in the respiratory or neurology departments. Owing to its rarity and the limited awareness of other departments regarding choriocarcinoma, the rate of misdiagnosis has increased significantly. In this report, lung metastasis occurred in 3 cases and all of which presented with typical respiratory symptoms (Table 1). One patient who presented with a massive pleural effusion and right lower atelectasis was referred to the respiratory department. The other complication was severe anemia (hemoglobin, 50.0 g/L). Blood transfusions and supportive treatment were administered, but the dizziness and headache worsened. CT of the brain revealed a metastasis. Case 3 was referred to the emergency department because of heavy vaginal bleeding, and physical examination revealed nodules on the vaginal wall. It is critical to be aware of the possibility of choriocarcinoma metastasis when puerperal females present with symptoms at common metastatic sites. Meanwhile, when choriocecinoma is considered, physical and imaging examinations of common metastatic sites are recommended, such as chest radiography and physical examination of the lungs. In addition, gynecological examinations should be performed to identify the bleeding sites.

In this report, 3 patients underwent vaginal delivery and 1 underwent a cesarean section. No pathological examination of the placenta was performed after delivery and the possibility of intraplacental choriocarcinoma (IC) could not be ruled out. IC commonly occurs in the third trimester of pregnancy with an atypical presentation, and only a few cases present with slight vaginal bleeding in the third trimester.^[14]^ Macroscopic examination of the placenta makes it difficult to detect abnormalities, and a few reports have found white nodular tissues surrounded by infarctions. However, diagnosis requires histopathological examination for confirm.^[15]^ Histopathological examination of the placenta is not routinely performed during a normal delivery. The incidence of reported IC is far lower than that in reality. Therefore, macroscopic examination of the placenta should be seriously emphasized, especially in high-risk groups, and histopathological examination should be performed when abnormalities are found.

The management of delayed postpartum hemorrhage depends on the cause, severity, and fertility requirements. Primary interventions include uterine massage, uterotonic drugs, and hemostasis, which can be controlled in most cases. Curettage is both a diagnostic and therapeutic tool that can help stop uterine bleeding and obtain biopsy specimens for histopathological examination. Embolization of the uterine arteries is performed, if necessary, or even hysterectomy, for refractory postpartum hemorrhage. Second, etiological treatment is critical. Choriocarcinoma is a gynecological tumor that can be completely cured by chemotherapy, and its therapeutic regimens are mainly based on clinical staging and prognostic scoring systems. A previous study reported that choriocarcinoma following term delivery resulted in a poorer prognosis than choriocarcinoma following other gestational events.^[9]^ The complete remission rate of postpartum choriocarcinoma has increased from 30% to 90% owing to advances in chemotherapy regimens.^[16]^ For the high-risk group that scored more than 6 on the World Health Organization prognostic scoring system, the therapeutic principle of postpartum choriocarcinoma was multidrug chemotherapy, assisted with surgery, and radiotherapy. EMA-CO regimen was the preferred regimen for high-risk group, with a complete remission rate of 80%.^[17]^ For patients who are refractory to EMA-CO or relapse from EMA-CO chemotherapy, salvage protocols, such as EP-EMA, bleomycin, etoposide, and cisplatin, 5-fluorouracil, actinomycin D, vincristine, and 5-fluorouracil, actinomycin D, etoposide and vincristine regimens, are recommended. In a previous retrospective study of 21 patients with resistance to the EMA-CO regimen, 16 of them (76%) achieved complete remission after switching to the EP-EMA regime or combined with surgery. EP-EMA regimens can be selected as the first-line treatment for extremely high-risk patients who score >12 in the World Health Organization prognostic scoring system. While these patients often cannot tolerate combination chemotherapy regimens, such as EP-EMA, a low-dose induction regimen can be administered first, such as the EP regimen (VP16 100 mg/m^2^ and DDP 20 mg/m^2^). Standardized combination chemotherapy can be administered after the patient’s general condition has improved. For patients with brain metastasis, the dose of MTX can be increased to 1 g/m^2^ to facilitate drug penetration into the blood-brain barrier. A previous study reported that MTX administered via intrathecal injection also achieved remission.^[18]^ Radiotherapy can also be used to achieve a better prognosis in patients with BM. Intracranial radiotherapy (20–30 Gy, 2 Gy/f) combined with high-dose MTX chemotherapy not only significantly decreased the metastatic foci in the brain but also increased MTX concentration within the cerebrospinal fluid and reduced the risk of cerebral hemorrhage.^[19]^

## 4. Conclusion

Even if choriocarcinoma is an extremely rare cause of delayed postpartum hemorrhage, it is important to be familiar with its differential diagnosis and individualized management. Obstetricians should be alert to the possibility of choriocarcinoma following full-term delivery and emphasize monitoring of β-hCG levels.

## Acknowledgments

We would like to thank all patients for their permission to provide their history and clinical data. This manuscript did not accept any financial assistance.

## Author contributions

**Conceptualization:** Guan-Lin Dai, Dan-Qing Wang.

**Data curation:** Guan-Lin Dai, Fu-Rong Tang.

**Funding acquisition:** Dan-Qing Wang.

**Supervision:** Yu Ma, Dan-Qing Wang.

**Writing – original draft:** Guan-Lin Dai, Dan-Qing Wang.

**Writing – review & editing:** Guan-Lin Dai, Yu Ma, Dan-Qing Wang.
